# Fake-cut maneuvers: biomechanical determinants of performance and ACL injury risk

**DOI:** 10.3389/fspor.2026.1856706

**Published:** 2026-06-22

**Authors:** Lasse Mausehund, Laura Bieri, Tron Krosshaug

**Affiliations:** 1 Oslo Sports Trauma Research Center, Department of Sports Medicine, Norwegian School of Sport Sciences, Oslo, Norway; 2 Department of Health Sciences and Technology, ETH Zürich, Zürich, Switzerland

**Keywords:** anterior cruciate ligament, change of direction, cutting biomechanics, knee joint loading, performance–injury conflict

## Abstract

**Background:**

Change-of-direction maneuvers can be executed with deceptive intent (‘fake-cuts’), yet the biomechanical determinants of fake-cut performance and their trade-offs with anterior cruciate ligament (ACL) injury risk remain poorly understood. Therefore, we compared cutting biomechanics between skilled and less skilled fake-cut performers during sport-specific fake-cut maneuvers.

**Methods:**

Ninety-six handball players performed fake-cuts while 3D kinematics and kinetics were recorded. Fake-cut performance was classified by each player's primary coach, and performance groups were compared using factorial ANOVA.

**Results:**

Skilled fake-cut performers displayed shorter ground contact times, higher movement speeds throughout stance, a shallower cutting angle and greater cutting width compared with less skilled performers. They also showed greater and faster head movements against the intended cutting direction, suggesting upper-body-driven deception. No clear group differences were observed in ACL injury risk–related knee biomechanics within the first 100 ms after initial contact. However, skilled performers showed greater peak knee abduction moment across the stance phase. Also, several biomechanical characteristics associated with superior fake-cut performance have previously been linked to greater ACL injury–related knee loading.

**Conclusion:**

Overall, effective fake-cut performance appears to rely on speed-oriented cutting mechanics and upper-body-driven deception. At the same time, the present findings may indicate a performance–injury conflict during fake-cut maneuvers. Cutting technique strategies that reduce ACL injury risk without impairing fake-cut performance warrant further investigation.

## Introduction

Change-of-direction (CoD) maneuvers are fundamental to performance in many team sports, including football, rugby, basketball, and handball. At the same time, they represent movement patterns during which non-contact anterior cruciate ligament (ACL) injuries commonly occur (1, 2). In many sport situations, CoD maneuvers are performed with deceptive intent to outmaneuver an opponent by deliberately misleading their perception of the intended movement direction (3). Such maneuvers, hereafter referred to as fake-cut maneuvers, typically involve an initial deceptive action—such as trunk, step, head, arm, or ball movement—intended to suggest one movement direction and provoke an opponent's response, followed by a rapid change of direction in another direction.

Previous studies have shown that fake-cuts differ from non-deceptive CoD tasks in torso, pelvis, and head kinematics, with fake-cuts showing exaggerated movements against the intended cutting direction — including pelvis rotation (4), torso lateral lean and rotation (3–5), and head lateral lean (4) and rotation (3). Such kinematic differences suggest that the deceptive component of fake-cuts may alter movement strategy compared with non-deceptive CoD tasks, with potential implications for both task performance and ACL injury risk.

Non-deceptive CoD performance is commonly quantified using task completion time (6, 7). Its biomechanical determinants have been extensively investigated, with faster CoD performance associated with shorter ground contact times, higher approach and exit velocities and greater lateral foot plant distances (7). Additional contributing factors include increased lower extremity joint moments and power, as well as greater trunk lean and rotation toward the intended cutting direction (7).

Fake-cut performance can be quantified using success rate, reflecting the ability to successfully deceive and pass an opponent. The determinants of fake-cut performance are multifaceted, as a successful execution of a fake-cut depends on a complex interaction between the physical, technical and perceptual-cognitive skills of both the attacker and the opponent, as well as differences in body mass and anthropometrics. Although a rapid CoD constitutes a key component of a fake-cut maneuver, fake-cut maneuvers extend beyond the CoD itself by incorporating deception. Consequently, some biomechanical factors associated with faster CoD performance, such as shorter ground contact times, may likely benefit fake-cut performance, whereas other factors, such as trunk lean and rotation towards the cutting direction, may be detrimental by compromising deception. This highlights the need for distinct investigations of fake-cut performance.

To our knowledge only one study has examined the biomechanical determinants of fake-cut performance. This study was limited to kinematic analyses of eight rugby players and concluded that a successful fake-cut requires a careful balance between exaggerated deceptive movements (i.e., torso and head rotation) and attenuated disguising strategies that enable rapid body reorientation toward the final direction [i.e., medial–lateral center of mass (COM) displacement and pelvis rotation] (3). However, the authors reported that the magnitude of individual biomechanical parameters alone was insufficient to explain the success of a deceptive action (3), highlighting the need for larger studies incorporating both kinematic and kinetic analyses.

A further important question is whether superior fake-cut performance may come at the cost of greater ACL injury–related knee loading. In non-deceptive CoD tasks, several biomechanical factors associated with faster CoD performance, such as higher approach velocities and larger lateral foot plant distances, have also been linked to increased ACL injury–related knee loading (8), suggesting a potential performance–injury conflict (6, 9). Whether a similar conflict may be present within fake-cut maneuvers is unknown. A deeper understanding of the performance aspects of fake-cut maneuvers is therefore needed to inform movement strategies that reduce injury risk without impairing fake-cut performance, particularly given that movement modifications perceived to compromise performance are unlikely to be adopted by athletes and coaches. Investigating this potential performance–injury conflict in females and in players with a previous ACL injury may be particularly important, given their elevated risk of ACL injury (10, 11).

To assess the biomechanical determinants of fake-cut performance, the primary aim of this study was to evaluate biomechanical differences between skilled and less skilled fake-cut performers during sport-specific fake-cut maneuvers. To address a potential performance–injury conflict during fake-cut maneuvers, we further assessed differences in ACL injury–related knee biomechanics between these groups. As a secondary aim, we examined whether these group differences varied according to sex or ACL injury status.

## Materials and methods

We used an exploratory cross-sectional design to investigate the effect of fake-cut performance skill on biomechanical characteristics during sport-specific fake-cut maneuvers. The data analyzed in the present study were collected as part of a larger experimental project examining the effects of sex and previous ACL injury on cutting biomechanics (*under review*).^1^

### Participants

We recruited female and male handball players with and without a previous ACL injury. Eligible participants were required to be at least 16 years of age and to be currently competing in one of the top four Norwegian divisions or the top junior division, or to have ceased participation within the previous year. Players were excluded if they had an acute musculoskeletal injury or reported pain at the time of testing. For those with a previous ACL injury, surgical reconstruction was required, at least one year had to have elapsed since surgery, and participants must have returned to match play. In total, 96 handball players were included in the study (Table 1).

**Table 1:** Participant characteristics of skilled and less skilled fake-cut performers (*n* = 96).

Variable	Category	Less skilled players (*n* = 47)	Skilled players (*n* = 49)	Adjusted MD (95% CI)	*p*-value (*η*_p_^2^)
Age		21.6 ± 3.5	21.3 ± 3.8	−0.3 (−1.9 to 1.2)	0.675 (<0.01)
Body mass (kg)		78.4 ± 11.6	76.7 ± 11.3	−1.2 (−4.7 to 2.4)	0.510 (<0.01)
Height (cm)		178.9 ± 8.8	178.1 ± 9.6	−1.0 (−3.7 to 1.6)	0.438 (0.01)
Sex	Female	24 (51%)	27 (55%)		
	Male	23 (49%)	22 (45%)		
Division^a^	Elite	4 (9%)	10 (20%)		
	First	11 (23%)	4 (8%)		
	Second	25 (53%)	20 (41%)		
	Third	5 (11%)	9 (18%)		
	Junior	2 (4%)	6 (12%)		
Currently competing		44 (94%)	48 (98%)		
Playing position	Back	30 (64%)	42 (86%)		
	Wing	13 (28%)	6 (12%)		
	Line	4 (9%)	1 (2%)		
Throwing arm	Right	41 (87%)	43 (88%)		
	Left	6 (13%)	6 (12%)		
Preferred cutting leg^a^	Right	13 (28%)	13 (27%)		
	Left	34 (72%)	36 (73%)		
Cutting frequency^a^	Rarely to occasionally	36 (77%)	10 (20%)		
	Often to very often	11 (23%)	39 (80%)		
Playing experience (yrs)		15.1 ± 3.9	14.7 ± 4.2	−0.4 (−2.1 to 1.3)	0.657 (<0.01)
Weekly training volume (minutes)	Handball practice	365.1 ± 104.6	364.2 ± 104.4	−12.5 (−56.5 to 31.4)	0.572 (<0.01)
	Game participation	42.7 ± 10.9	40.2 ± 12.5	−2.9 (−8.0 to 2.1)	0.251 (0.01)
	Strength training	222.4 ± 106.2	208.8 ± 113.0	−15.0 (−62.2 to 32.2)	0.530 (<0.01)
Previous ACL injury	No	25 (53%)	33 (67%)		
	Yes	22 (47%)	16 (33%)		
Time since ACL surgery (months)		44.0 ± 27.7	38.8 ± 22.4	5.2 (−11.3 to 21.7)	0.524 (0.20)

Values are mean ± SD for continuous variables and *n* (%) for categorical variables.

MD, mean difference; η_p_^2^, partial eta squared effect size; ACL, anterior cruciate ligament.

a Coach-reported.

All participants provided written informed consent prior to participation. The study was approved by the ethics committee of the Norwegian School of Sport Sciences (approval number 328–130324) and the Norwegian Agency for Shared Services in Education and Research, and conducted in accordance with the latest revision of the Declaration of Helsinki.

### Test procedure

All participants attended a single test session in a biomechanical laboratory. A total of 72 retro-reflective markers were attached to each participant. Marker positioning was based on a previously published protocol (12), with identical placement of all lower-limb and trunk markers. To improve feasibility, four markers were omitted from each upper extremity (epicondyles, lateral shoulder, and hand), and the two wrist-condyle markers were replaced with a single marker placed on the dorsal aspect of the wrist. To ensure consistency, all markers were placed by the same trained physical therapist. Participants wore their own indoor court shoes and handball shorts, with female players additionally wearing a sports bra. After completing a static calibration trial and a standardized warm-up, participants proceeded to the experimental tasks.

**Figure 1:**
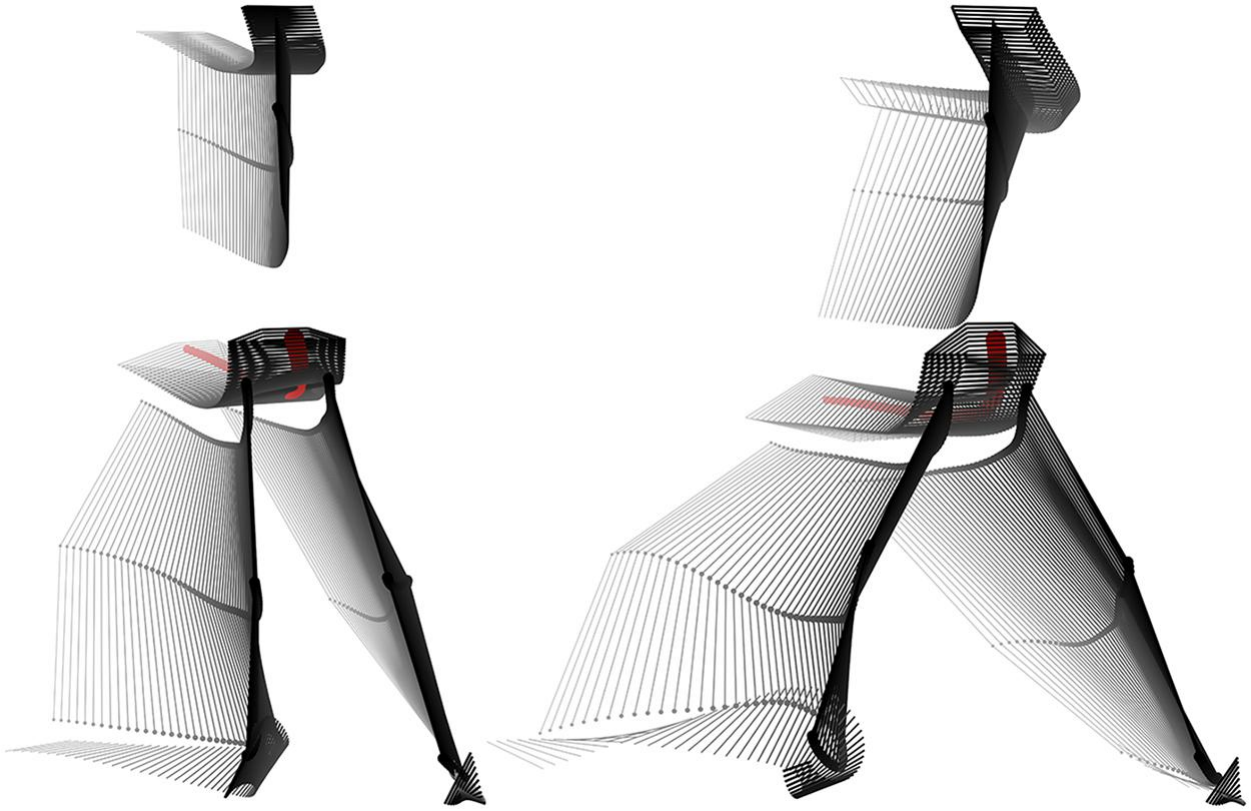
Representative stick-figure illustration of the cutting kinematics of two individual players—a poor (left) and a very good (right) fake-cut performer—executing a right-leg cut towards the left (viewed from behind). The black stick-figure indicates initial contact and gradually fades to grey at toe-off. The red dot represents the center of mass.

Participants performed a handball-specific fake-cut maneuver with maximal, match-like effort. The task was designed to simulate a typical one-versus-one attacking situation in handball. Players first passed the ball to a teammate positioned at the sideline, then approached a static human defender from straight ahead with a 6-m run-up, received the ball immediately before executing the cutting maneuver, attempted to outmaneuver and pass the defender, and completed the sequence by feigning a shot on goal. The fake-cut consisted of using deceptive body movements to suggest movement in one direction, followed by a rapid change of direction past the defender in the opposite direction. The specific cutting technique, deceptive action, cutting angle, and approach speed were not prescribed, allowing players to perform the maneuver as they would in a real match situation. This unrestricted task design was intentional, as the aim was to identify the movement strategies that skilled and less skilled fake-cut performers naturally adopt during a sport-specific, match-like cutting maneuver. Players were required to contact a single force plate with the cutting leg while remaining unaware of its presence, thereby ensuring that attention was directed toward outmaneuvering the defender. Missed contacts were addressed by small adjustments to the defender's position in subsequent trials to increase the likelihood of valid force-plate contact, while players remained unaware of the force plate. Although the specific cutting technique was not prescribed, nearly all players executed the maneuver using a split-step (Figure 1), with only a few performing a side-step (13). Three valid trials were recorded for each leg, with leg order randomized. Kinematic and kinetic data were collected for all trials. The use of three trials was based on previous reliability work showing that adequate reliability for sport-specific cutting biomechanics can be achieved from three trials, with only minor improvements when additional trials are included (14).

### Performance classification

Following the testing session, each player's primary handball coach was contacted to provide an experience-based evaluation of that player's fake-cut performance. In total, ratings were obtained from 65 unique coaches. Coaches reported the player's preferred cutting leg (left or right) and the frequency with which the player performed cutting maneuvers using the preferred leg on a 5-point Likert scale (1 = never, 5 = very frequent). Coaches then rated their player's fake-cut performance by answering the following question: ‘*How good is your player at outmaneuvering and passing a defender using a fake-cut maneuver, compared to other players in the same division?’* Responses were provided on a 5-point Likert scale (1 = very poor, 2 = poor, 3 = average, 4 = good, 5 = very good) and referred to the player's preferred cutting leg. The specific Norwegian wording of the questions was developed in close collaboration with experienced handball coaches and players to ensure that the terminology reflected sport-specific handball language and was understood as intended by the respondents. Coach ratings were used to classify fake-cut performance, reflecting applied expertise and prolonged observation in competitive settings, and thereby enhancing ecological validity. These ratings were subsequently used to assign players to performance-based groups.

### Instrumentation and data processing

All measurements were acquired synchronously using a 16-bit analog-to-digital conversion board (USB-2533; Measurement Computing Corporation, Norton, MA, USA) and integrated to Qualisys Track Manager (version 2023.3; Qualisys AB, Gothenburg, Sweden). Data were subsequently processed and analyzed in MATLAB (version R2024b; MathWorks Inc., Natick, MA, USA). Three-dimensional kinematic data were collected at 200 Hz using 23 optical cameras (Oqus 400 and 700; Qualisys AB, Gothenburg, Sweden). Ground reaction force (GRF) and center of pressure data were recorded at 1000 Hz from two floor-mounted force plates (AMTI LG6-4-1; Watertown, MA, USA).

Kinematic and kinetic data were low-pass filtered using a second-order Butterworth digital filter applied bidirectionally (zero-lag), with a 20 Hz cut-off frequency. Ankle and knee joint centers were defined as the midpoints between the malleoli and epicondyle markers, respectively. Hip joint center locations and pelvic coordinate systems were established according to the methods described by Bell et al. (15) and Seidel et al. (16). Segment inertial properties were estimated using regression equations reported by de Leva (17). Resultant external joint moments of the lower extremity were calculated within a rigid-body framework using the explicit formulation described by Hof (18). All model calculations were performed using a previously described MATLAB implementation (19). For each trial, the stance phase was defined as the interval during which the unfiltered vertical GRF exceeded 30 N.

### Outcome measures

Our primary outcome measures were selected based on their relevance to non-deceptive CoD performance, deceptive actions, and ACL injury risk. Specifically, these included ground contact time, horizontal COM speed, cutting width (similar to lateral foot plant distance), lower-extremity peak joint moments, and head and torso kinematics. Peak KAM within the first 100 ms of stance was considered the primary ACL injury–related outcome.

Performance-related outcome measures included cutting technique variables and lower-extremity 3D joint moments and angles extracted at defined events [initial contact [IC], peak, and toe-off [TO]] across the entire stance phase. Calculation methods for these variables have been described previously (20, 21). Torso and head kinematics were defined relative to the global coordinate system, reflecting the perspective of the defender. Torso and head lateral lean were defined relative to a vertical axis.

ACL injury–related outcome measures were extracted within the first 100 ms of stance and included 3D peak knee joint moments and 3D knee joint angles at IC and at their peak values. To isolate their contributions to peak knee abduction moment (KAM), the frontal-plane knee moment arm (20) (normalized to leg length) and the corresponding resultant GRF at the time of the peak KAM were also extracted. The first 100 ms of stance was selected because non-contact ACL injuries typically occur shortly after IC (22). For each participant, the average of three trials was used for analysis.

### Data analysis and statistics

No player was classified as having very poor fake-cut performance, resulting in four performance groups: poor (*n* = 8), average (*n* = 39), good (*n* = 35), and very good (*n* = 14). To ensure sufficiently large group sizes and more robust estimation of interaction effects, the primary statistical analyses were conducted using two performance groups. Accordingly, the poor and average performance groups were combined to form the *less skilled* group, whereas the good and very good performance groups were combined to form the *skilled* group. To provide additional context beyond these primary comparisons, we conducted exploratory analyses using all four performance categories. Each player's preferred cutting leg was used in all analyses.

All statistical analyses were conducted in MATLAB (version R2024b; MathWorks Inc., Natick, MA, USA). Assumptions were assessed using residual Q–Q plots for normality, residuals-versus-fitted plots for homoscedasticity, and Levene's test for homogeneity of variance across groups. To compare cutting biomechanics between skilled and less skilled fake-cut performers, a factorial analysis of variance (ANOVA) was conducted with performance group, sex, and ACL injury status as fixed factors. Sex and ACL injury status were included to adjust the performance-group comparisons for these potential confounding factors and to examine whether performance-group differences varied according to sex or ACL injury status. Significant two-way interactions (i.e., performance   ×   sex and performance   ×   ACL injury status) were followed up with simple-effects analyses. Main effects of sex and ACL injury status were outside the scope of the present study and were therefore not interpreted. Exploratory analyses were performed using unadjusted Welch ANOVA to explore differences in cutting biomechanics across all four performance groups. Significant ANOVA results were followed up with *post hoc* tests.

The level of significance was set at *p* ≤ 0.05. Results are reported as mean ± SD, along with adjusted mean differences (MD) and 95% confidence intervals (CI). Effect sizes are reported as partial eta squared (*η*_p_^2^) and generalized eta squared (*η*^2^_G_), and interpreted using conventional thresholds as small (0.01), medium (0.06), and large (0.14) (23, 24).

## Results

Differences between skilled and less skilled fake-cut performers across the entire stance phase are presented in Tables 2, 3. Skilled fake-cut performers executed the fake-cut maneuver with shorter ground contact times, a shallower cutting angle, and higher movement speed throughout the stance phase. In addition, skilled players showed greater cutting width and a more externally rotated hip and foot position at IC. They also exhibited greater peak head lateral lean and greater head lateral lean velocity at IC against the cutting direction than less skilled players.

**Table 2:** Cutting technique variables over the entire stance phase in skilled and less skilled fake-cut performers (*n* = 96).

Variable	Less skilled players (*n* = 47)	Skilled players (*n* = 49)	Adjusted MD (95% CI)	*p*-value (η_p_^2^)
Ground contact time (ms)	287.5 ± 34.2	273.3 ± 31.1	−14.2 (−28.1 to −0.4)	**0.044** (**0.05)**
Cutting angle (°)	50.6 ± 11.6	46.0 ± 8.2	−4.7 (−8.9 to −0.5)	**0.028** (**0.05)**
Approach speed at IC (m·s^−1^)	2.99 ± 0.36	3.22 ± 0.40	0.21 (0.06 to 0.35)	**0.005** (**0.09)**
Minimum speed (m·s^−1^)	2.36 ± 0.38	2.59 ± 0.37	0.23 (0.08 to 0.38)	**0.004** (**0.09)**
Exit speed at TO (m·s^−1^)	3.19 ± 0.36	3.37 ± 0.36	0.17 (0.04 to 0.31)	**0.012** (**0.07)**
Sideways COM velocity at IC (m·s^−1^)^a^	−0.10 ± 0.34	0.03 ± 0.30	0.12 (−0.01 to 0.26)	0.077 (0.04)
Sideways COM velocity at TO (m·s^−1^)^a^	2.36 ± 0.32	2.41 ± 0.26	0.04 (−0.05 to 0.13)	0.397 (0.01)
Vertical COM velocity at IC (m·s^−1^)	−1.44 ± 0.31	−1.43 ± 0.22	0.02 (−0.09 to 0.12)	0.726 (<0.01)
Vertical COM velocity at TO (m·s^−1^)	0.43 ± 0.18	0.38 ± 0.19	−0.03 (−0.11 to 0.04)	0.398^d^ (0.01)
Cutting width at IC (°)	21.9 ± 2.6	23.1 ± 2.1	1.1 (0.1 to 2.0)	**0.034** (**0.05)**
Cutting depth at IC (°)	22.1 ± 5.4	20.9 ± 4.7	−1.0 (−3.2 to 1.1)	0.328 (0.01)
Foot strike angle at IC (°)^b^	−6.7 ± 15.2	−6.5 ± 14.4	0.4 (−6.0 to 6.7)	0.908 (<0.01)
Foot progression angle at IC (°)^c^	−7.5 ± 9.5	−13.5 ± 8.9	−6.2 (−9.9 to −2.4)	**0.002** (**0.11)**
Torso lateral lean at IC (°)^a^	−3.2 ± 6.3	−3.9 ± 6.9	−0.3 (−3.1 to 2.5)	0.831 (<0.01)
Torso lateral lean minimum (°)^a^	−14.5 ± 6.5	−16.7 ± 8.2	−2.2 (−5.1 to 0.7)	0.127 (0.03)
Torso lateral lean velocity at IC (°·s^−1^)^a^	−70.7 ± 65.2	−87.4 ± 67.5	−22.3 (−46.0 to 1.5)	0.066 (0.04)
Torso rotation at IC (°)^a^	−4.9 ± 12.4	−6.9 ± 12.2	−1.3 (−6.0 to 3.3)	0.570 (<0.01)
Torso rotation minimum (°)^a^	−11.8 ± 8.9	−12.5 ± 11.3	−0.5 (−4.6 to 3.5)	0.794 (<0.01)
Torso rotation velocity at IC (°·s^−1^)^a^	−121.8 ± 99.2	−122.2 ± 110.1	−4.5 (−46.2 to 37.1)	0.830 (<0.01)
Head lateral lean at IC (°)^a^	−3.3 ± 5.3	−3.8 ± 6.0	0.1 (−2.3 to 2.5)	0.942 (<0.01)
Head lateral lean minimum (°)^a^	−12.4 ± 8.8	−16.5 ± 9.5	−4.1 (−7.5 to −0.7)	**0.020** (**0.06)**
Head lateral lean velocity at IC (°·s^−1^)^a^	−39.0 ± 61.2	−60.5 ± 76.1	−23.4 (−46.7 to −0.2)	**0.048** (**0.04)**
Head rotation at IC (°)^a^	7.7 ± 9.8	4.8 ± 8.6	−1.7 (−5.6 to 2.2)	0.380^d^ (0.01)
Head rotation minimum (°)^a^	4.8 ± 10.4	0.6 ± 12.5	−3.4 (−8.2 to 1.4)	0.161 (0.02)
Head rotation velocity at IC (°·s^−1^)^a^	1.2 ± 60.1	−6.8 ± 58.2	−10.7 (−34.6 to 13.3)	0.378 (0.01)

Values are mean ± SD. MD = mean difference; η_p_^2^ = partial eta squared effect size; IC = initial contact; TO = toe-off; COM = center of mass.

a Positive values indicate movement toward the intended cutting direction.

b Negative values indicate rearfoot strike.

c Negative values indicate foot external (outward) rotation.

d Significant performance   ×   ACL injury status interaction. Main effects should therefore be interpreted with caution. Simple effects are reported in the text.

Significant effects (*p* ≤ 0.05) are shown in **bold**.

**Table 3:** Three-dimensional kinematics and kinetics over the entire stance phase in skilled and less skilled fake-cut performers (*n* = 96).

Variable	Less skilled players (*n* = 47)	Skilled players (*n* = 49)	Adjusted MD (95% CI)	*p*-value (η_p_^2^)
Hip flexion angle at IC (°)	48.6 ± 8.2	50.5 ± 7.6	2.3 (−1.1 to 5.8)	0.174 (0.02)
Hip flexion angle peak (°)	54.0 ± 8.5	54.7 ± 7.6	0.8 (−2.7 to 4.2)	0.650 (<0.01)
Hip abduction angle at IC (°)	20.2 ± 6.1	21.0 ± 6.6	1.1 (−1.5 to 3.7)	0.391 (0.01)
Hip abduction angle peak (°)	40.2 ± 5.4	42.5 ± 4.5	2.0 (0.0 to 4.1)	**0.048** (**0.04)**
Hip internal rotation angle at IC (°)	1.1 ± 6.3	−1.4 ± 5.3	−2.7 (−5.0 to −0.4)	**0.021** (**0.06)**
Hip internal rotation angle peak (°)	10.2 ± 6.5	8.8 ± 6.3	−1.7 (−4.2 to 0.8)	0.186 (0.02)
Knee flexion angle at IC (°)	24.3 ± 7.8	26.9 ± 8.3	3.0 (−0.3 to 6.4)	0.071 (0.04)
Knee flexion angle peak (°)	56.6 ± 5.2	56.2 ± 4.8	−0.3 (−2.5 to 1.8)	0.761 (<0.01)
Knee abduction angle at IC (°)	0.6 ± 3.5	1.6 ± 3.7	0.9 (−0.5 to 2.4)	0.212 (0.02)
Knee abduction angle peak (°)	9.7 ± 6.3	11.4 ± 7.3	1.5 (−1.1 to 4.2)	0.241 (0.02)
Knee internal rotation angle at IC (°)	8.2 ± 7.9	7.6 ± 7.5	−0.8 (−4.0 to 2.4)	0.619 (<0.01)
Knee internal rotation angle peak (°)	20.0 ± 5.1	18.9 ± 4.7	−1.4 (−3.3 to 0.6)	0.167 (0.02)
Ankle dorsiflexion angle at IC (°)	−7.5 ± 11.2	−7.5 ± 11.0	0.1 (−4.6 to 4.7)	0.981 (<0.01)
Ankle dorsiflexion angle peak (°)	15.4 ± 5.4	13.6 ± 6.9	−1.8 (−4.4 to 0.8)	0.168 (0.02)
Hip flexion moment peak (Nm·kg^−1^)	3.17 ± 0.65	3.15 ± 0.79	0.03 (−0.26 to 0.32)	0.843 (<0.01)
Hip adduction moment peak (Nm·kg^−1^)	1.31 ± 0.57	1.47 ± 0.52	0.15 (−0.06 to 0.37)	0.148 (0.02)
Hip internal rotation moment peak (Nm·kg^−1^)	1.76 ± 0.55	1.84 ± 0.48	0.10 (−0.10 to 0.30)	0.338 (0.01)
Knee flexion moment peak (Nm·kg^−1^)	3.31 ± 0.66	3.34 ± 0.61	−0.01 (−0.26 to 0.23)	0.917 (<0.01)
Knee abduction moment peak (Nm·kg^−1^)	1.00 ± 0.39	1.23 ± 0.53	0.24 (0.04 to 0.44)	**0.021** (**0.06)**
Knee internal rotation moment peak (Nm·kg^−1^)	0.38 ± 0.21	0.42 ± 0.23	0.04 (−0.05 to 0.13)	0.409 (0.01)
Ankle dorsiflexion moment peak (Nm·kg^−1^)	2.26 ± 0.42	2.27 ± 0.47	0.02 (−0.16 to 0.20)	0.843 (<0.01)
Resultant GRF peak (N·kg^−1^)	29.76 ± 4.30	30.07 ± 3.06	0.26 (−1.32 to 1.84)	0.740 (<0.01)

Values are mean ± SD.

MD, mean difference; η_p_^2^, partial eta squared effect size; IC, initial contact; GRF, ground reaction force.

Variable names indicate the positive direction. External joint moments are reported.

Significant effects (*p* ≤ 0.05) are shown in **bold**.

No significant differences between skilled and less skilled fake-cut performers were observed for knee biomechanics associated with ACL injury risk within the first 100 ms after IC (Table 4). However, peak knee abduction angle and peak KAM tended to be higher in skilled than less skilled performers, with borderline significance (*p* = 0.078 and *p* = 0.083, respectively). Across the whole stance phase, peak KAM was significantly greater in skilled players (Table 3).

**Table 4:** ACL injury–related knee kinematics and kinetics within the first 100 ms after initial contact in skilled and less skilled fake-cut performers (*n* = 96).

Variable	Less skilled players (*n* = 47)	Skilled players (*n* = 49)	Adjusted MD (95% CI)	*p*-value (η_p_^2^)
Knee flexion angle peak (°)	55.3 ± 4.4	55.2 ± 4.2	0.0 (−1.8 to 1.9)	0.987 (<0.01)
Knee abduction angle peak (°)	6.9 ± 4.9	9.3 ± 6.8	2.2 (−0.3 to 4.6)	0.078 (0.03)
Knee internal rotation angle peak (°)	17.4 ± 5.6	17.3 ± 4.8	−0.4 (−2.5 to 1.7)	0.711 (<0.01)
Knee flexion moment peak (Nm·kg^−1^)	3.27 ± 0.65	3.33 ± 0.62	0.01 (−0.23 to 0.25)	0.915 (<0.01)
Knee abduction moment peak (Nm·kg^−1^)	0.74 ± 0.41	0.95 ± 0.59	0.19 (−0.03 to 0.41)	0.083 (0.03)
Knee internal rotation moment peak (Nm·kg^−1^)	0.36 ± 0.22	0.37 ± 0.25	0.00 (−0.10 to 0.10)	0.995 (<0.01)
Resultant GRF at peak KAM (N·kg^−1^)	21.03 ± 7.39	21.36 ± 6.35	0.50 (−2.32 to 3.31)	0.727 (<0.01)
Knee moment arm at peak KAM (ratio)	0.031 ± 0.019	0.038 ± 0.026	0.006 (−0.004 to 0.016)	0.216 (0.02)

Values are mean ± SD.

ACL, anterior cruciate ligament; MD, mean difference; η_p_^2^, partial eta squared effect size; GRF, ground reaction force; KAM, knee abduction moment. Variable names indicate the positive direction. External joint moments are reported.

No significant interactions between performance group and sex were observed, indicating that the biomechanical differences between skilled and less skilled fake-cut performers did not clearly differ between female and male players. Significant performance   ×   ACL injury status interactions were identified only for vertical COM velocity at TO (*p* = 0.047, *η*_p_^2^ = 0.04) and head rotation at IC (*p* = 0.048, *η*_p_^2^ = 0.04). Among injury-free players, skilled performers showed lower vertical COM velocity at TO than less skilled performers [MD = −0.11 m·s⁻^1^, 95% CI (−0.20, −0.01), *p* = 0.025] and greater head rotation against the cutting direction at IC [MD = −5.7°, 95% CI (−10.6, −0.8), *p* = 0.023], whereas no differences were observed among ACL-injured players.

Exploratory analyses across all four performance groups revealed significant overall group effects for sideways COM velocity at IC (*p* = 0.026, *η*^2^_G_ = 0.11), sideways COM velocity at TO (*p* = 0.020, *η*^2^_G_ = 0.09), minimum torso lateral lean (*p* = 0.005, *η*^2^_G_ = 0.05), and torso rotation velocity at IC (*p* = 0.007, *η*^2^_G_ = 0.03). *post hoc* analyses showed that players classified as poor fake-cut performers exhibited lower sideways COM velocity at IC than the average, good, and very good performance groups [MDs = 0.30 to 0.43 m·s^−1^, 95% CI (0.03, 0.72)] and lower sideways COM velocity at TO compared with the average and good groups [MDs = 0.30 to 0.33 m·s^−1^, 95% CI (0.08, 0.54)]. Poor performers also demonstrated less peak torso lateral lean against the cutting direction than all other groups [MDs = −5.1 to −6.5°, 95% CI (−11.1, −1.0)], as well as lower torso rotation velocity against the cutting direction at IC than average and good performers [MDs = −63.2 to −72.0°·s^−1^, 95% CI (−115., −17.2)]. Representative examples of cutting kinematics for poor and very good fake-cut performers are shown in Figure 1 and Supplementary Video S1.

## Discussion

The purpose of this study was to examine biomechanical differences between players who are skilled and less skilled at executing game-specific fake-cut maneuvers, while also exploring a potential performance–injury conflict related to ACL injury risk. The main findings showed that skilled fake-cut performers adopted a distinct cutting strategy characterized by shorter ground contact times, higher movement speeds throughout stance, a shallower cutting angle, and greater cutting width. In addition, they exhibited more pronounced and faster head movements against the cutting direction. Together, these movement strategies appear to enhance deceptive effectiveness and overall fake-cut performance. No clear group differences were observed in ACL injury risk–related knee biomechanics within the first 100 ms after IC, although peak KAM across the stance phase was greater in skilled performers. The observed performance group differences were largely consistent across sex and ACL injury status.

The biomechanical differences between skilled and less skilled fake-cut performers were similar in female and male players and in players with and without previous ACL injury, as evidenced by the near absence of interaction effects. This is important because sex-related differences in neuromuscular and biomechanical characteristics during cutting tasks have previously been reported (25, 26). However, in the present study, no performance   ×   sex interactions were observed, suggesting that the biomechanical characteristics distinguishing skilled from less skilled fake-cut performers did not clearly differ between female and male players. Similarly, only two performance   ×   ACL injury status interactions were observed, both with small effect sizes. These findings suggest that fake-cut performance skill was associated with broadly similar biomechanical characteristics irrespective of sex and ACL injury status. Importantly, this does not imply that sex or ACL injury status are unimportant for cutting biomechanics or previous ACL injury risk, but rather that they did not appear to meaningfully modify the performance-group differences observed in the present study.

### Biomechanical determinants of fake-cut performance

Skilled fake-cut performers exhibited 5% shorter ground contact times (small effect size), 5%–10% higher movement speeds throughout stance (medium effect sizes) and 1° greater cutting width (small effect size) than less skilled performers (Table 2), suggesting that rapid redirection and speed-oriented cutting mechanics are central to successful fake-cut execution. The same biomechanical factors have also been associated with faster non-deceptive CoD performance (7, 27), indicating that fake-cut performance partly depends on general CoD ability.

However, some differences appeared to be specific to fake-cut performance, particularly its deceptive component. Whereas contributing factors to faster CoD performance include greater trunk lean and rotation toward the cutting direction, as well as greater hip internal rotation angles and internal foot progression angles (7), the opposite pattern was observed for superior fake-cut performance—namely, greater hip and foot external rotation and, in the exploratory analyses, greater torso lateral lean and torso rotation velocity against the cutting direction. Trunk lean and rotation against the cutting direction may be important for deception during a fake-cut, whereas a more externally rotated lower-limb orientation may allow rapid redirection without revealing the final direction too early.

Head movements against the intended cutting direction also distinguished skilled from less skilled fake-cut performers. Skilled players exhibited 4° greater peak head lateral lean and 61% greater head lateral lean velocity against the cutting direction than less skilled players (moderate and small effect sizes, respectively; Table 2). In addition, injury-free skilled players rotated their head 6° more against the cutting direction at IC than injury-free less skilled players. Although torso kinematics did not differ in the primary two-group analysis, exploratory analyses suggested that higher-performing players exhibited 5–7° greater peak torso lateral lean and 102%–116% higher torso rotation velocity at IC against the cutting direction than poor fake-cut performers (Figure 1). Together, these findings suggest that upper-body movements, particularly of the head, contribute meaningfully to deceptive effectiveness during fake-cut maneuvers. The discrepancy between the primary and exploratory torso findings may indicate that a minimum level of torso movement and torso movement velocity against the intended cutting direction is important for successfully outmaneuvering an opponent, whereas further increases do not necessarily provide additional benefit. This interpretation is consistent with Brault et al. (3), who proposed that successful deception requires a careful balance between disguise and deceit strategies, constrained within thresholds that preserve movement credibility.

In our exploratory analysis, poor fake-cut performers demonstrated a small sideways COM velocity at IC directed away from the intended cutting direction, potentially reflecting an attempt to fake toward the opposite side, whereas good and very good performers already directed their COM slightly toward the intended cutting direction at IC. This may suggest that poor performers prioritize overt deception by initially directing their COM toward the false direction, whereas higher performing players rely more on upper-body cues for deception while initiating earlier COM redirection toward the cutting direction to enable a rapid change of direction.

Coinciding with Brault et al. (3), we observed 2° greater peak hip abduction angles and 1° larger cutting widths at IC in skilled players (small effect sizes; Tables 2, 3). While Brault et al. (28) proposed that this exaggeration serves to deceive the opponent, we agree with the argumentation of Warren-Westgate et al. (4) that a more laterally displaced foot primarily facilitates rapid COM redirection through increased medial–lateral force and impulse generation. This interpretation is also consistent with our exploratory findings showing that higher-performing players exhibited 14%–16% higher sideways COM velocity at TO compared with poor fake-cut performers.

Collectively, the present findings suggest that effective fake-cut performance may be supported more by movement speed than by executing a sharper cut, while deception may be conveyed primarily through upper-body kinematics. Practitioners should emphasize short ground contact times, high approach, minimum, and exit speeds, and a sufficiently wide cutting width to facilitate rapid COM redirection. Deceptive intent appears to be more effectively conveyed through controlled and fast head and torso movements against the cutting direction, rather than through initial COM movement toward the false direction. A subtle early bias in COM velocity toward the intended direction may facilitate a more rapid CoD once deceptive cues have been presented. Training interventions may therefore benefit from integrating speed-oriented cutting biomechanics with controlled upper-body deception, although increasing approach speed may require prior development of strength, coordination, and control.

### Performance-injury conflict

Our findings may indicate a potential performance–injury conflict during fake-cut maneuvers. Although these differences were not significant, skilled fake-cut performers demonstrated 2° greater peak knee abduction angles (*p* = 0.078) and 26% greater peak KAM (*p* = 0.083) within the first 100 ms after IC compared with less skilled performers (small effect sizes; Table 4); variables which have been associated with ACL injury mechanisms (22) and increased ACL loading (29) and injury risk (30, 31), respectively. Across the whole stance, peak KAM was significantly 24% greater in skilled performers, with a moderate effect size. Together, these findings suggest that some of the biomechanical characteristics associated with superior fake-cut performance may also contribute to greater frontal-plane knee loading. This interpretation is strengthened by the fact that several variables associated with superior fake-cut performance in the present study have previously been linked to increased ACL injury–related knee loading, including higher approach speed, greater cutting width and hip abduction, and increased torso lateral lean and rotation against the cutting direction (8, 20).

These conflicting factors further highlight the challenge of reducing ACL injury-related knee loading without compromising performance. However, several modifiable variables previously associated with greater ACL injury-related knee loading were not associated with superior fake-cut performance in the present study, including a sharper cutting angle, greater knee abduction angle, a rearfoot strike pattern, greater vertical COM velocity at IC, greater internal hip rotation and foot progression angles, and smaller knee flexion angles (8, 21). These variables may therefore represent promising targets for reducing injury-related knee loading without impairing fake-cut performance and are discussed in more detail below.

Although a shallower cutting angle was associated with superior fake-cut performance in the present study, a minimum cutting angle is necessary to execute an effective fake-cut. Further reducing the cutting angle may jeopardize performance, rendering this factor unsuitable as a target for injury-risk reduction. Also, the optimal cutting angle is likely to vary depending on the specific playing situation.

Reducing knee abduction angles during fake-cut maneuvers may represent an effective strategy to decrease KAM without compromising performance. Notably, knee abduction angle has been identified as the primary contributor to KAM during fake-cut maneuvers (20) and can be effectively modified using simple verbal cues, resulting in reduced KAM (32). Reducing vertical COM velocity at IC by avoiding ‘jumping’ into the cut also appears to be a promising strategy for decreasing KAM (32) and was not associated with fake-cut performance in the present study.

Increasing foot strike angle at IC—i.e., landing on the midfoot or forefoot rather than the rearfoot—has also been shown to decrease KAM (32) and, based on the present findings, is unlikely to negatively impact performance. However, we acknowledge previously raised concerns (33) that a forefoot landing strategy may increase ankle instability and, thus, ankle injury risk. While forefoot landing increases gastrocnemius involvement (34), which may contribute to anterior tibial shear and ACL loading, it has also been associated with increased hamstrings activation (34) that may partially counteract this ACL loading. Importantly, the foot strike pattern may partially be dictated by the breaking demands of the fake-cut maneuver. Factors such as higher approach speeds and sharper cutting angles necessitate greater braking forces, thereby promoting more anterior foot placement relative to the COM, making a rearfoot strike more natural, especially when the foot is in a neutral or externally rotated position. Conversely, under lower braking demands—which may also reduce ankle stability demands—a midfoot or forefoot strike may represent a viable alternative for decreasing KAM while maintaining performance. Specifically, this likely applies only to the early impact absorption phase, with the foot firmly planted during the main part of the cut (32).

Since greater internal hip rotation and foot progression angles have been associated with increased KAM (8), reducing these factors may represent a viable strategy for decreasing KAM. Notably, a more externally rotated hip and foot position at IC was associated with superior fake-cut performance in the present study. Previous research examining the effects of modifying foot progression angle on KAM has reported conflicting effects, with some studies observing no effect (35, 36) and others reporting both increases (37) and decreases (38) in KAM with external rotation. However, external rotation has been consistently associated with reduced knee abduction angles and increased knee flexion angles (35, 37, 38) — attributes considered protective with respect to ACL injury risk (30, 39). Therefore, adopting a neutral to slight external rotation of the hip and foot (≤20°) at IC may represent a viable strategy to reduce ACL injury risk without compromising performance.

Finally, smaller knee flexion angles at IC have been associated with larger KAM (8) and greater anterior tibial shear forces (40), and smaller peak knee flexion has been directly linked to increased ACL injury risk (39). In the present study, knee flexion angle was not related to fake-cut performance. However, previous research on CoD performance indicates that greater knee flexion ROM is associated with longer ground contact times and slower performance times (9, 41). Hence, shifting the knee flexion ROM toward greater flexion angles without increasing overall knee flexion ROM —thereby maintaining lower-limb stiffness—may represent a potential strategy to reduce injury risk without compromising performance. Such an approach would, however, require greater knee extensor strength and power to maintain rapid force production during the cutting maneuver. Notably, a slight increase in knee flexion ROM and ground contact time may not necessarily impair fake-cut performance, suggesting some flexibility in this trade-off.

Collectively, to reduce ACL injury-related knee joint loading without compromising fake-cut performance, cutting technique strategies should focus on reducing knee abduction angles, adopting a mid- to forefoot landing strategy under lower braking demands, minimizing vertical COM velocity at IC by avoiding ‘jumping’ into the cut, adopting a neutral to slight external rotation of the hip and foot at IC, and shifting knee flexion ROM toward greater flexion angles. These technique considerations appear relevant across sexes and irrespective of ACL injury status, and may be particularly important for females and for players with a previous ACL injury, given their elevated risk of ACL injury. Future research should investigate whether modifying these variables reduces KAM during fake-cut maneuvers without negatively affecting performance-related outcomes.

While these recommendations for fake-cut maneuvers mostly align with those proposed to reduce ACL injury risk during non-deceptive CoD tasks without affecting completion time (6, 9, 33), important differences exist. Most notably, greater torso lateral flexion and rotation toward the intended cutting direction are commonly recommended to reduce injury risk and enhance CoD performance (6, 9, 33). However, the present findings suggest that such strategies may be less compatible with effective fake-cut maneuvers, in which deceptive performance appears to depend on movement patterns directed opposite the intended cutting direction. This highlights the need for future research to clearly distinguish between non-deceptive CoD tasks and fake-cut maneuvers and to adopt task-specific performance metrics, as fake-cut performance should not be evaluated solely based on completion time or ground contact time.

### Strengths and limitations

To our knowledge, this is the largest (*n* = 96) and most comprehensive study to date examining fake-cut performance. However, a limitation of the present study is that players were recruited from different competitive divisions. Although this factor may influence cutting biomechanics and fake-cut performance, its relatively even distribution across performance groups is assumed to have mitigated its potential confounding effect.

An advantage of the present performance classification approach is that fake-cut performance was evaluated at a general player level by each player's coach, rather than being determined by success against a specific laboratory defender. This reduces the influence of the skill level, positioning, or behavior of an individual defender and allows a clearer focus on attacker-specific movement strategies. However, coach-based evaluation may introduce subjective classification bias or halo effects and did not allow assessment of inter-rater reliability. Future studies would benefit from objective match-based performance metrics, such as the percentage of successful fake-cuts during competitive play, ideally combined with ratings from multiple coaches. In addition, fake-cut success in match play is likely determined by a multifactorial interaction between biomechanical, physical, technical, and perceptual-cognitive factors of both the attacker and defender. Future studies should therefore integrate multivariable approaches including attacker–defender interactions, strength and power measures, and perceptual-cognitive factors.

Movements preceding IC were not assessed in the present study. Although such movements may influence fake-cut performance, particularly its deceptive component, they are likely less relevant during split-step cutting maneuvers, as body position at IC is relatively neutral (Figure 1). Preceding movements may play a more prominent role in shuffle-based side-step techniques, which are more commonly employed in sports such as rugby (28).

The present study focused on discrete biomechanical variables averaged across three fake-cut trials. However, ACL injury risk may not be fully explained by instantaneous peak loading alone, as repetitive loading and cumulative ligament fatigue mechanisms may also contribute to tissue failure (42).

Finally, it is important to acknowledge that there is no single optimal cutting technique applicable to all athletes. Optimal cutting technique is likely both individual-specific—depending on factors such as anthropometrics and strength—and context-specific, depending on match situations such as the number and positioning of defenders and ball location. Consequently, movement variability and a large movement repertoire may be key to successful cutting performance across varying conditions.

## Conclusion

Players who are skilled at executing game-specific fake-cut maneuvers adopt a distinct cutting technique strategy characterized by shorter ground contact times, higher movement speeds throughout stance, a shallower cutting angle and greater cutting width compared with less skilled performers. Superior fake-cut performance was also associated with more pronounced and faster head movements against the intended cutting direction and, in exploratory analyses, greater and faster torso movements in that direction, highlighting the importance of upper-body kinematics for deception. Together, these findings suggest that superior fake-cut performance depends on a combination of speed-oriented cutting mechanics and deceptive upper-body movement. At the same time, several biomechanical characteristics associated with superior fake-cut performance in the present study have previously been linked to greater ACL injury–related knee loading, and skilled performers also exhibited greater peak KAM across the stance phase. These findings may therefore indicate a potential performance–injury conflict during fake-cut maneuvers. Sex and ACL injury status did not appear to meaningfully influence the observed group differences. Future research should determine whether fake-cut performance can be maintained while reducing ACL injury–related knee loading.

## Data Availability

The raw data supporting the conclusions of this article will be made available by the authors, without undue reservation.

## Footnotes

^1^Mausehund L, Mai P, Bill K, Krosshaug T. Females unload the knee more than males after ACL injury during sport-specific cutting maneuvers. in review.

## Supplementary material

Supplementary Video S1 Representative stick-figure animation of the cutting kinematics of two individual players—a poor (left) and a very good (right) fake-cut performer—executing a right-leg cut towards the left (viewed from behind). The whole stance phase, from initial contact to toe-off, is shown. The red dot represents the center of mass.
